# Indigenous women’s experiences about the pregnancy-puerperal cycle

**DOI:** 10.1590/0034-7167-2023-0410

**Published:** 2024-10-11

**Authors:** Lubiane Boer, Francisca Georgina Macedo de Sousa, Rizioléia Marina Pinheiro Pina, Margarita Poblete, Léris Salete Bonfanti Haeffner, Dirce Stein Backes

**Affiliations:** I Universidade Franciscana. Santa Maria, Rio Grande do Sul, Brazil.; II Universidade Federal do Maranhão. São Luís, Maranhão, Brazil.; III Universidade Federal do Amazonas. Manaus, Amazonas, Brazil.; IV Universidad Católica del Maule. Talca, Chile.

**Keywords:** Qualitative Research, Indigenous Culture, Pregnancy, Postpartum Period, Primary Health Care, Investigación Cualitativa, Cultura Indígena, Embarazo, Periodo Posparto, Atención Primaria de Salud

## Abstract

**Objectives:**

to understand the experiences of indigenous women regarding the pregnancy-puerperal cycle.

**Methods:**

qualitative, exploratory and descriptive research, carried out between May and August 2023 with 27 pregnant women from Indigenous Villages in Mato Grosso, Brazil, through open individual interviews. The data was analyzed using Reflexive thematic analysis.

**Results:**

data analysis resulted in the following themes: Cultivation of labor and birth in its natural and sacred path; Unique practices and beliefs associated with breastfeeding; Evolved or reductive thinking? The participants suggest inviolable practices and beliefs, which must be welcomed, respected and enhanced by indigenous health teams.

**Final Considerations:**

the experiences of indigenous women regarding the pregnancy-puerperal cycle are unique and motivated by inviolable cultural and religious beliefs, which transcend scientific knowledge, certainties and the linearity of contemporary approaches, normally established as order.

## RESUMEN

**Objetivos:**

comprender las experiencias de mujeres indígenas del ciclo embarazo-puerperio.

**Métodos:**

se trata de una investigación cualitativa, de carácter exploratorio y descriptivo, realizada entre mayo y agosto de 2023 con 27 gestantes de Aldeas Indígenas de Mato Grosso, Brasil, mediante entrevistas individuales abiertas. Los datos se analizaron bajo la óptica del análisis temático del tipo reflexivo.

**Resultados:**

el análisis de los datos dio lugar a los siguientes temas: Culto del parto y nacimiento en su curso natural y sagrado; Prácticas y creencias únicas asociadas a la lactancia materna; ¿Pensamiento evolucionado o reduccionista? Los participantes sugieren prácticas y creencias inviolables, que deben ser acogidas, respetadas y potenciadas por los equipos de salud indígenas.

**Consideraciones Finales:**

las experiencias de las mujeres indígenas en el ciclo embarazo-puerperio son singulares y están motivadas por creencias culturales y religiosas inviolables que trascienden el conocimiento científico, las certezas y la linealidad de los enfoques contemporáneos, que suelen establecerse como orden.

## INTRODUCTION

Among the Sustainable Development Goals (SDGs) is “Health and well-being”, in order to ensure access to quality health and the promotion of well-being for everyone at all ages^(1)^. To achieve this objective, the United Nations (UN) defined specific goals, including reducing the maternal mortality rate by 2030 to less than 70 deaths per 100,000 live births, in addition to achieving universal health coverage, including access to quality essential health services. The Federal Brazilian Government, in partnership with the Institute of Applied Economic Research (Ipea), in order to make them more relevant to local challenges, adapted the SDG/Agenda 2030 Goals to the national reality. Thus, SDG 3.1 was redefined and aims to reduce the maternal mortality ratio to a maximum of 30 deaths per 100,000 live births^(2)^. Failure to meet targets related to reducing maternal deaths may be an indication of the ineffectiveness of public policies aimed at this population^(3)^. However, how can this goal be achieved among indigenous women, who experience specific cultural processes?

The pregnancy-puerperal cycle translates into an affirmative and strengthening experience in the existential course of women, but it can also incur adverse events and result in maternal and infant mortality. From this perspective, maternal and child health is considered a global priority and one of the essential public health services for achieving the SDGs. However, in this process of discussions and assistance alignments for pregnant and postpartum women, it is necessary to consider customs, beliefs and practices that are culturally accepted and significant for each population group^(4, 5)^.

In indigenous cultures, in particular, motherhood, childbirth and birth have a unique meaning and with specificities guaranteed in the United Nations Declaration on the Rights of Indigenous Peoples (UNDRIP), a comprehensive international instrument that endorses the human rights of indigenous people. This Declaration guarantees indigenous peoples the right to maintain their health practices, religious and medicinal practices, and qualified access to health services, without any discrimination. These concessions must be ensured by States, through local strategic measures and policies^(6, 7, 8)^.

Maternal and child health management and assistance arrangements among indigenous peoples are, in addition to being unique, also complex, as they involve historical experiences of segregation, experimental procedures, specific and often mutilating interventions. Indigenous pregnant women who a few decades ago gave birth in their villages, supported by midwives with specific knowledge, began to experience medical and hospital interventions motivated by hegemonic health knowledge. The role of the indigenous midwife, perceived as a vocational function, with a sacred and continuous meaning for the mother and the baby, in the family, began to be assumed by health professionals^(9, 10, 11)^.

Although guaranteed in specific government policies and defined in Axis 13 - Indigenous Health and Axis 14 - Maternal and Child Health of the Ministry of Health’s Research Priorities Agenda^(12)^, the health rights of indigenous women remain with little protection and, sometimes , impact irreparable paths in the pregnancy-puerperal cycle and with exposure to different risks. Among the challenges to be overcome, qualified access to prenatal and postpartum care, the intercultural qualification of professionals, among other challenges related to social and health determinants and conditions stand out^(13, 14, 15)^.

In recent years, initiatives have been taken to indigenize healthcare and put on the agenda the specificities guaranteed by UNDRIP and the SDGs, whose goals can be achieved through the implementation of approaches based on rights and culturally sensitive indigenous practices. The research question is: What are the experiences of indigenous women regarding the pregnancy-puerperal cycle?

## OBJETICVES

To understand the experiences of indigenous women regarding the pregnancy-puerperal cycle.

## METHODS

### Ethical aspects

During the research, the recommendations of Resolutions of the National Health Council (CNS) No. 466/2012 and Operational Norm No. 001 of 2013 of the CNS were considered. The research project was approved by the National Ethics and Research Commission and Free and Informed Consent was obtained from all participants involved in this study in writing. To maintain anonymity, the participants’ statements were identified throughout the text with the letter ‘G’, for Pregnant, followed by a number corresponding to the order of the statements: G1...G27.

### Study Design

Qualitative, exploratory and descriptive research. Its approach has meanings that make it possible to expand perspectives, beliefs, convictions and specific cultural experiences that cannot be reduced to specific, linear and fragmented variables. The Consolidation Criteria for Qualitative Research Reports (COREQ) was followed^(16)^.

### Methodological Procedures

#### Study Setting

The corpus of this study was composed of 27 pregnant women, living in villages in the municipality of Querência, Mato Grosso, Brazil, which has a total of 31 Indigenous Villages, with approximately 5,738 villages and distinct socioeconomic, racial, ethnic, cultural and geographic specificities. Participants were accessed for convenience, through local health teams and, after accepting participation, they were contacted personally by the researchers for further clarification and to schedule a day and time for data collection.

The study included indigenous pregnant women from any of the 31 villages, age, language, gestational period and in physical and emotional conditions to participate in the interviews on the days and times previously scheduled. Pregnant women speaking other languages unaccompanied by an interpreter (family member, Indigenous Health Agent, Midwife or other) are excluded from the study. The interviews were audio recorded using a cell phone and then transcribed for analysis.

#### Data collection and organization

Data were collected between the months of May and August 2023, through individual interviews, lasting an average of 20 minutes. The open individual interviews were conducted based on the following questions: Tell me about your pregnancy, prenatal care, childbirth, birth and breastfeeding. In your opinion, what could be different in the care provided to pregnant women? These questions were widely explored, in each of the topics, in order to expand perceptions, experiences and practices specific to indigenous culture.

The interviews, previously scheduled, were conducted by a researcher with expertise in indigenous health management and a collaborating nurse who works in indigenous villages. Previously, meetings of pregnant women were held at the Municipal Health Department, in a reserved room decorated with handcrafted objects specific to indigenous culture, such as: coas, necklaces, hammocks, baskets, straws, moringas and others and, subsequently, they were individual interviews were carried out. The interviews were carried out in the same place, after meeting pregnant women.

#### Data analysis

The data were analyzed based on the Reflexive thematic analysis technique^(17)^. In this analysis process, emphasis was placed on the cultural significance of each statement and experience. In addition to counting the number of information, we sought to understand the existential and cultural meaning of each statement expressed and/or not by the indigenous pregnant woman.

Reflexive thematic analysis was adopted in this study in order to enable the systematic recording of experiences and insights, in addition to providing a spontaneous and flexible coding of the unique meanings attributed by indigenous pregnant women. Thus, this analysis was systematized into six sequential and complementary phases: Familiarization based on repeated readings of the data and a draft list of ideas; Generation of initial codes, manually, by systematizing relevant extracts; Search for themes based on the classification of different codes; Refinement of themes based on validation of initial themes; Naming the themes based on the essence that each theme represents in the set of codes; and the Production of the report that offered a reflective description of what was experienced(16), from the perspective of the SDG.

## RESULTS

**Figure 1:**
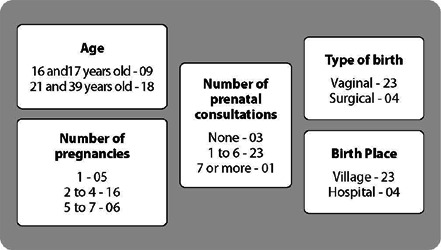
Age and data related to pregnancy, prenatal care and childbirth of indigenous women

Initially, the characterization of the study participants is presented, in relation to the variables age, number of prenatal consultations, number of pregnancies, type and place of birth, as shown in Figure 1.

The Reflexive data analysis resulted in three reflective themes, namely: Cultivation of labor and birth in their natural and sacred path; Unique practices and beliefs associated with breastfeeding; Evolved or reductive thinking?

### Cultivation of labor and birth in its natural and sacred path

Natural childbirth is strongly desired and defended in indigenous culture. This practice is reproduced from mother to daughter and so on. They recognize the body as sacred and, therefore, must be free from cuts or physical aggression. In the same way, “prayers” or any religious practice is accepted and reproduced as a blessing and divine protection. “Cuts, tied legs and arms” are associated with hospital births (cesarean section) and have a connotation of detachment, coldness and violence. Therefore, they want to give birth in the most natural way possible and with their family members.

*I want it like the village, I don’t want them to cut it so my child can be born, I also don’t want them to tie my legs and arms like I’ve heard they do in hospital.* (G3)

*I want to stay here, close to my mother. I don’t want them to cut me.* (G5)

*It’s better in the village, because here there’s a midwife who helps me, there’s my mother who helps me too, there’s a doctor and staff.* (G13)

*The midwife was there, she prayed and helped me so he was born, then she helped me clean up and I went to lie in the hammock, because I was feeling weak.* (G12)

Childbirth and birth in indigenous culture are filled with a mixed feelings, rituals and beliefs that contribute to and determine birth in its most natural form possible. In indigenous culture, “baby time” is respected, regardless of the waiting time for its birth. The baby’s time is respected and perceived as unique and sacred, that is, as an inviolable path that can be manipulated by external forces.

*It’s different from here in the village. In there* [in hospital], *they don’t wait for the baby to be born, they put serum so the baby is born faster and that’s not normal for us, here we wait for the baby to be born, I didn’t like that.* (G5)

*I had my first child when I was 16, through a C-section. He weighed 4kg. The doctor said that my belly was too big and that he wouldn’t be born normally. Then when the pain started, the doctor told me he would remove it so it would go away faster. It was difficult to accept.* (G15)

It was evident in several statements that even when advised by the health team, indigenous pregnant women prefer to give birth in the village, due to the fact that they can count on the presence of their mother, mother-in-law, family midwife and other people close to them.

*They were born in the village, in the hammock, I was with my mother and my mother-in-law who is the midwife. I felt a lot of pain, but they helped me.* (G7)

*In the village there’s my mother, there’s my family, there’s a midwife and they help us, the baby was made and it won’t stay in the womb forever, one day it will come out, at the right time, and they help us deliver the babies in village.* (G11)

*I don’t want to go to the city* [hospital] *to give birth. I want to stay here with my children and my family, my mother and my sister.* (G24)

In other statements, indigenous pregnant women demonstrated that birth in the hospital is safer, sometimes due to induction by the health team and other times for unrelated reasons related to obstetric complications. In one statement, in particular, it was evident that the pregnant woman wanted the birth to take place in the hospital, although she was advised to give birth in the village by her family.

*I wanted to be living in the city so the baby could be born in the city* [hospital]. *It’s safer, there’s a doctor. I have a son who is disabled.* (G1)

*I want him to be born in the hospital, because there is a doctor there, and it’s better, but my mother wants him to be born in the village. Here in the village it’s very far and I sometimes feel very weak.* (G11)

*When I was well, with food and walking, the child could be born in the village, but today as I’m in a lot of pain, there are days when the pain comes, and there are days when I’m very weak, so I think it’s better to be born in the city.* (G16)

*The team was monitoring me in the village and I had a problem with changes in blood pressure, my foot and body were swollen and I was very heavy, so I couldn’t give birth here, so I had to give birth in the hospital, guided by the area doctor and together with the other women. The midwife said I had to deliver in the city and there the birth wasn’t progressing and I had to have a cesarean section, everything went well.* (G20)

*The two girls were born in the village and the two boys were born in the hospital, because I felt pain from urine infection and then you can’t earn money in the village.* (G25)

In one statement, in particular, a certain empowerment of the pregnant woman was noted in relation to the autonomous choice of the type of birth, even though it took place in the hospital. Even though she was in the hospital, she was welcomed in her decision.

*As I already had a cesarean section and wanted the second child to be a natural birth, so as not to cut my belly, I had to go to the city. When I arrived at the hospital I said that I didn’t want a cesarean, that I wanted a normal birth. Then I went to the delivery room and he was born normal and well.* (G24)

It was learned from the participants’ statements that natural childbirth has an unquestionable meaning in indigenous culture. In this journey, above all, the child’s autonomy is respected, that is, their time and birth time, even if the mother has to wait many hours for the birth. The mother, in turn, submits to the long hours of pain, welcoming birth as sacred and inviolable. Religious practices, such as prayers, teas, impositions and others, adopted by midwives, constitute a powerful aid in relieving labor pain.

### Unique practices and beliefs associated with breastfeeding

The study participants, in most cases, reproduced conceptions culturally expressed in indigenous environments, devoid of autonomous thought. Although very limited and, at times, evasive, the participants’ statements were limited to reproducing expressions coming from their mothers or someone linked to the family, such as: She said that the child is supposed to be healthy. (G9) She said it gets stronger this way. (G15) She said it can’t be like that. (G17)

The practice of breastfeeding is unquestionable in indigenous culture. At no point did the participants mention weak milk or the impossibility of breastfeeding. Only one pregnant woman mentioned having little milk, but she managed it with specific medicinal herbs. Another reported abdominal pain and needed support to be able to breastfeed.

*He still breastfeeds. He is 2 years old. And now when this other baby is born, one needs to stop.* (G7)

*Everyone sucked. I think that’s what matters most.* (G9)

*Yes, in one of them I almost had no milk, so I took grass and my milk soon came. He became strong, his bones were strong, he didn’t get as many diseases.* (G18)

*Yes, I always breastfed, once it took a while because my belly hurt* [caesarean section] *and then my mother helped me. I was in pain for many days, only then at home it was better.* (G25)

It was evident, in most of the statements, that the indigenous pregnant woman stops breastfeeding her child, upon the occasion of a new birth. Although breastfeeding is a culturally accepted and encouraged practice, most women reported weakness, tiredness, discouragement and, in two cases, the occurrence of depression and not wanting to get out of bed.

*I felt pain, weakness. There are days when I don’t want to get up, my husband and I have wanted to have a baby.* (G2)

*She’s tired of having babies. Complains a lot about weakness.* (G5)

*I don’t want it anymore, I feel very weak when I’m pregnant. It’s bad for sleeping and the baby moves a lot.* (G11)

It was identified, in other statements, that although subjugated in their culture, indigenous women recognize their cultural strengths and consider them superior to those of white people, especially when comparing breast milk. In his understanding, artificial milk is associated with the disease as follows:

*Because breast milk makes the child very strong, because white people’s milk is different from yours, because then I don’t need to give white people’s milk.* (G11)

*Because with breast milk the baby grows strong and healthy and in the village there is no white milk. I gave the milk until the other was born.* (G16)

The other children were breastfed. Just a disabled person who uses white people’s milk. (G22)

*Yes, because breast milk keeps them well and they grow. The milk you buy, white milk, is only for those who have an illness.* (G25)

In almost all of the statements, the participants are devoid of autonomy, that is, the autonomy of the indigenous pregnant woman is linked to cultural processes, fully legitimized and accepted. Therefore, the right to choose is ensured by the strength of culture and, above all, by the perpetuation of religious beliefs and practices loaded with meaning at a personal and collective level. Thus, there is a paradox between what is understood and defended by autonomy from an intercultural perspective and complexity thinking.

### Evolved or reductive thinking?

When asked what could be better or different in relation to assistance to pregnant and postpartum women, the answers were, for the most part, superficial and evasive, but loaded with a historical and cultural meaning.

*I wouldn’t know.* (G2, G5, G9, G10, G15, G18, G20)

*I don’t know how to answer.* (G1, G3, G6, G19, G22, G23)

*I don’t know.* (G7, G9, G12, G16, G24, G25, G27)

In some statements, the desire to improve prenatal care within the villages was evident, so that there is no need to transfer to hospitals, due to long distances. In general, all pregnant women want to give birth in the village, close to their families and, in this sense, they suggest more doctors, medications and tests, as follows:

*Have more tests, have medicine and a doctor in the village.* (G8)

*Have a vaccine, have medicine to help. Also have a doctor in all villages.* (G11)

*Take tests in the village and get more medicine.* (G13)

*More doctors and more staff in the area to serve our family.* (G14)

*Have more medicine, more vitamins for pregnant women and for when their children get sick.* (G17)

*Having a team that stays in the village so that when we have a small baby, we can help them when they are sick.* (G21)

*There could be a midwife to deliver the baby in the village and the doctor too.* (G26)

Although superficial and evasive in their speeches, the study participants demonstrated experiences and knowledge that are unique to them and, by no means, inferior or superior to the knowledge of any other culture. And, in this sense, researchers ask themselves: is this an evolved or reductive thought? How to dialogue with this plural and intercultural diversity of knowledge? Where do we need to evolve and how do we evolve so as not to incur new mutilations and achieve the goals defined in the SDGs?

## DISCUSSION

Access to prenatal care and qualified care during childbirth and the postpartum period are closely related to the organization and structure of Primary Health Care, as well as being associated with the reduction of inequalities and the promotion of social justice. These guidelines are also close to the principles of the Unified Health System (SUS), by conceiving equity, universality and comprehensiveness in health care as a goal for achieving better results at the individual and collective level^(18)^.

The Ministry of Health defines that prenatal care must begin by the Ί2th week of pregnancy and consist of at least six follow-up consultations. Inadequate or inefficient prenatal care is a determining factor for unfavorable outcomes in labor and birth, as well as a risk factor for maternal and child mortality^(19)^, especially among indigenous women. Thus, the high fertility of indigenous women, represented by short gestational intervals and the occurrence of teenage pregnancy are generally factors that influence the pregnancy-puerperal cycle and compromise the goal set out in the 2030 Agenda.

The pregnancy-puerperal cycle encompasses, in itself, a mix of feelings, beliefs and (de)constructions, most of the time nonlinear and predictable in the eyes of modern science, which requires answers, at any cost, to different existential events. In indigenous culture, events, even if adverse, occur naturally and with cultural specificities that need to be considered, especially in childbirth and birth, considered natural and sacred events. Any advancement in this area demands rapprochement, dialogue and sharing of experiences that are culturally accepted and valued among indigenous women^(20)^.

In the speech of the participants in this study, an important paradox was evident when they discussed the “indigenous village”, an environment that is familiar and welcoming to them, and the “hospital”; an impersonal, cold and distant environment, deciphered as a city. This “hospital” environment hurts their body, the sacred, as a result of the cuts caused by the cesarean section that pregnant women do not want. This same “hospital” environment distances them from their mother, mother-in-law, midwife and other family members who, in the villages, participate and celebrate birth, considered a collective event.

Studies^(6, 21, 22)^ endorse this thinking of the participants, by emphasizing that in many indigenous cultures, birth consists of a religious ceremony, in which a new life is celebrated, that is, an event that celebrates the passage from the spiritual world to the physical world. Birth ceremonies are directly linked to the earth, the sun, the sky and, therefore, the closer they are to family and culture, the more sacred and promising the child’s health and future will be. Recognizing the place of birth and honoring its sacredness constitutes a significant strategy to ensure cultural identity and implement indigenous rights.

Another unique and complex phenomenon is associated with the moment of birth, the “baby time” and “mother time”. As it is a natural and sacred journey, the baby’s time is always respected in indigenous culture, even if the mother has to wait long hours for the birth of her child. In this relationship, cessation, as it involves intervention, is seen as a violation of the sacred, of the “sacred body” that carries a sacred being. The body needs to be free from external interventions or interference.

Studies demonstrate, in this direction, that in indigenous culture women prefer natural births and strive for traditional and conventional practices, that is, births in villages and in the company of families. These traditional practices promote mother-baby interaction and reduce complications associated with perineal ruptures or infections. These practices are supported by midwives who have skills based on experience and knowledge acquired informally through traditions and practices associated with indigenous communities^(23, 24)^.

Indigenous theory has its own worldview, related to traditions and closely interconnected with all created things. Therefore, we speak of a holistic and comprehensive theory, which involves past, present and future and includes spiritual, emotional, mental and physical elements of being and moving. From this perspective, the “time of birth of the baby” is the time predestined by the spirits of heaven and by Creation^(25, 26)^.

Another important paradox is related to “breastfeeding” and “white people’s milk”. Breast milk is loaded with energy that binds, strengthens, brings together and perpetuates the sacred among indigenous generations. “White people’s milk” is related to the disease – a sick child who, for some reason, was born by cesarean section and in a hospital environment. Studies demonstrate, from this perspective, that in indigenous culture the health-disease process is closely related to immediate breastfeeding and, equally, associated with natural childbirth in villages, as a sacred and protected phenomenon^(27, 28)^.

In the indigenous context, breastfeeding is not only a source of nutrition for the child, but constitutes security, protection and connection between mother/family and child, motivated by cultural and spiritual values, which bring the child closer and place the child at the center. of the existential process. Studies^(29, 30, 31)^ suggest a broad understanding of these cultural, social, community and collective aspects, in order to enhance favorable breastfeeding experiences among indigenous women. For breastfeeding to continue to be a culturally accepted practice, it is essential that specific policies be implemented for indigenous women, considering that they breastfeed their children until the birth of their next child and, as a result, report weakness, discomfort and discomfort.

The results of this study showed that indigenous women demonstrate the need to expand the presence of doctors in the villages, in addition to the provision of medicines and exams, in order to facilitate immediate access to care for special cases. However, studies developed in other countries showed that indigenous women prefer to be assisted and guided by indigenous health professionals, due to their solid understanding of culturally accepted and encouraged practices^(32, 33)^. In this sense, the same studies demonstrate that indigenous health agents provide holistic solutions and support targeted to the needs of each woman and family.

The pregnancy-puerperal cycle requires, on the part of health professionals who work in indigenous villages, a singular and multidimensional look, that is, woven by a set of threads/elements that evoke, at least, more than one circumstance or associative possibility for form culturally significant knowledge^(34)^ and, thus, contribute to achieving the goals established by the 2030 Agenda. To this end, it is necessary to strip away prejudiced perceptions and uncover references that expand theoretical perspectives and the induction of approaches that consider the uniqueness and multidimensionality of each indigenous woman/family.

The pregnancy-puerperal cycle in indigenous culture dates back to a historical-hegemonic tradition marked by normative and prescriptive relationships, in which scientific knowledge overlapped with lived cultural experience. In this relationship, the indigenous pregnant or postpartum woman was subjugated to prescriptive biomedical knowledge, deprived of her knowledge and meaning of life and care. This hegemonic superpower, consolidated in modernity, resulted in a cultural void and divided the indivisible and the separate parts, making them without a meaning of life and without identity^(34)^.

Advancing access to and quality of care for indigenous women (pregnant and postpartum women) requires ruptures in hegemonic thinking and an expanded and systemic understanding of different cultural realities. (Re)signifying and reconnecting culturally relegated elements to the background require a prospective perspective on the part of health professionals in general. The 2030 Agenda and the Ministry of Health’s Agenda of Priorities will be achieved by resuming values and principles of life, health and well-being of different cultures.

### Study limitations

A limitation of this study is the non-generalization of the results, considering that the interviews were carried out with indigenous pregnant and postpartum women from only one region of Brazil, which has continental geographic dimensions and cultural specificities that vary from village to indigenous village.

### Contributions to the area of Nursing and Health

The contributions of this study to the advancement of Nursing and Health science are associated with the perception that it is necessary to reconnect elements of indigenous culture, fragmented and simplified by the supremacy of hegemonic knowledge in health. In this process of reframing, valuing and enhancing resources specific to indigenous culture, the Nurse can appear as a protagonist and inducer of prospective policies, capable of contributing to the achievement of the Sustainable Development Goals.

## FINAL CONSIDERATIONS

The experiences of indigenous women regarding the pregnancy-puerperal cycle are unique and motivated by inviolable cultural, environmental and religious beliefs, which transcend scientific knowledge, certainties and the linearity of contemporary approaches, normally established as order in the context of health.

The pregnancy-puerperal cycle in indigenous culture needs to be welcomed and understood in its multiple connections and in association with environmental and transcendental phenomena. Childbirth and birth must be considered as a natural event and in its sacred and collective dimension. The practice of breastfeeding is not only a source of nutrition for the child, but a source of energy, safety, protection and connection between mother/family and child.
