# Supplementary material for: Indigenous women’s experiences about the pregnancy-puerperal cycle
Source: Rev Bras Enferm. 2024 Oct 11;77(Suppl 2):e20230410. doi: 10.1590/0034-7167-2023-0410 (PMC11468837; doi:10.1590/0034-7167-2023-0410)
Supplement: Translation

# Translation: Vivências de mulheres indígenas acerca do ciclo gravídico-puerperal

**DOI:** 10.1590/0034-7167-2023-0410pt

**Authors:** Lubiane Boer, Francisca Georgina Macedo de Sousa, Rizioléia Marina Pinheiro Pina, Margarita Poblete, Léris Salete Bonfanti Haeffner, Dirce Stein Backes

**Affiliations:** I Universidade Franciscana. Santa Maria, Rio Grande do Sul, Brasil.; II Universidade Federal do Maranhão. São Luís, Maranhão, Brasil.; III Universidade Federal do Amazonas. Manaus, Amazonas, Brasil.; IV Universidad Católica del Maule. Talca, Chile.

**Keywords:** Pesquisa Qualitativa, Cultura Indígena, Gravidez, Puerpério, Atenção Primária à Saúde

## Abstract

**Objetivos:**

compreender as vivências de mulheres indígenas acerca do ciclo gravídico-puerperal.

**Métodos:**

pesquisa qualitativa, de caráter exploratório e descritivo, realizada entre maio e agosto de 2023 com 27 gestantes de Aldeias Indígenas do Mato Grosso, Brasil, por meio de entrevistas individuais abertas. Os dados foram analisados pela análise temática do tipo *Reflexive.*

**Resultados:**

a análise dos dados resultou nas temáticas: Cultuação do parto e nascimento em seu percurso natural e sagrado; Práticas e crenças singulares associadas ao aleitamento materno; Pensamento evoluído ou redutor? As participantes sugerem práticas e crenças invioláveis, as quais devem ser acolhidas, respeitadas e potencializadas pelas equipes de saúde indígena.

**Considerações Finais:**

as vivências de mulheres indígenas acerca do ciclo gravídico-puerperal são singulares e motivadas por crenças culturais e religiosas invioláveis, as quais transcendem o saber científico, as certezas e a linearidade das abordagens contemporâneas, normalmente, instituídas como ordem.

## INTRODUÇÃO

Dentre os Objetivos de Desenvolvimento Sustentável (ODS) tem-se a “Saúde e bem-estar”, de modo a assegurar o acesso à saúde de qualidade e a promoção do bem-estar para todos em todas as idades^(1)^. Para o alcance deste objetivo, a Organização das Nações Unidas (ONU) definiu metas específicas dentre as quais a de reduzir a taxa de mortalidade materna até 2030 para menos de 70 mortes por 100 mil nascidos vivos, além de atingir a cobertura universal de saúde incluindo o acesso a serviços de saúde essenciais de qualidade. O Governo Federal, em parceria com o Instituto de Pesquisa Econômica Aplicada (Ipea), no intuito de torná-las mais pertinentes aos desafios locais, adaptou as Metas dos ODS/Agenda 2030 à realidade nacional. Dessa forma, o ODS 3.1 foi redefinido e visa reduzir a razão de mortalidade materna para no máximo 30 mortes por 100 mil nascidos vivos^(2)^. O não cumprimento de metas relacionadas à redução dos óbitos maternos pode ser indício da ineficácia das políticas públicas direcionadas para este público^(3)^. Como, no entanto, alcançar essa meta entre as mulheres indígenas, as quais vivenciam processos culturais específicos?

O ciclo gravídico-puerperal traduz uma experiência afirmativa e fortalecedora no curso existencial de mulheres, mas pode também incorrer em eventos adversos e resultar na mortalidade materna e infantil. Sob esse enfoque, a saúde materno-infantil é considerada prioridade global e um dos serviços essenciais de saúde pública para o alcance dos ODS. Nesse processo de discussões e alinhamentos assistenciais às gestantes e puérperas é preciso considerar, todavia, costumes, crenças e práticas culturalmente aceitas e significativas para cada grupo populacional^(4, 5)^.

Nas culturas indígenas, em especial, a maternidade, o parto e o nascimento possuem um significado singular e com especificidades garantidas na Declaração das Nações Unidas sobre os Direitos dos Povos Indígenas (UNDRIP), instrumento internacional abrangente que endossa os direitos humanos dos povos indígenas. Essa Declaração assegura aos povos indígenas o direito de manter as suas práticas de saúde, condutas religiosas e medicinais, acesso qualificado aos serviços de saúde, sem qualquer discriminação. Essas concessões devem ser asseguradas pelos Estados, por meio de medidas e políticas estratégicas locais^(6, 7, 8)^.

Os arranjos de gestão e assistência em saúde materno-infantil entre os povos indígenas são, além de singulares, também complexos, por envolverem experiências históricas de segregação, procedimentos experimentais, intervenções pontuais e, frequentemente, mutilantes. As gestantes indígenas que há poucas décadas davam à luz em suas aldeias, apoiadas pelas parteiras detentoras de conhecimentos específicos, passaram a vivenciar intervenções médico-hospitalares motivadas pelo saber hegemônico na saúde. O papel da parteira indígena, apreendido como função vocacionada, com sentido sagrado e continuado à mãe e o bebê, na família, passou a ser assumido por profissionais de saúde^(9, 10, 11)^.

Embora garantidos em políticas governamentais específicas e definidos na Eixo 13 - Saúde Indígena e Eixo 14 - Saúde Materno Infantil da Agenda de Prioridades de Pesquisa do Ministério da Saúde^(12)^, os direitos à saúde das mulheres indígenas seguem com pouca proteção e, por vezes, impactam em percursos irreparáveis no ciclo gravídico-puerperal e com exposição a riscos distintos.

Destacam-se, dentre os desafios a serem superados, o acesso qualificado aos cuidados pré-natais e pós-parto, a qualificação intercultural dos profissionais, dentre outras apostas relacionadas aos determinantes e condicionantes sociais e de saúde^(13, 14, 15)^.

Nos últimos anos, vem sendo tomadas iniciativas para indigenizar os cuidados de saúde e colocar em pauta as especificidades garantidas pela UNDRIP e pelos ODS, cujas metas podem ser alcançadas mediante a implementação de abordagens baseadas nos direitos e nas práticas indígenas culturalmente sensíveis. Tem-se como questão pesquisa: Quais as vivências de mulheres indígenas acerca do ciclo gravídico-puerperal?

## OBJETIVOS

Compreender as vivências de mulheres indígenas acerca do ciclo gravídico-puerperal.

## MÉTODOS

### Aspectos éticos

Considerou-se, no percurso de pesquisa, as recomendações da Resoluções do Conselho Nacional de Saúde (CNS) nº 466/2012 e Norma Operacional nº 001 de 2013 do CNS. O projeto de pesquisa foi aprovado pela Comissão Nacional de Ética e Pesquisa e o Consentimento Livre e Esclarecido foi obtido de todos os participantes envolvidos deste estudo por meio escrito. Para manter o anonimato, as falas das participantes foram identificadas, ao longo do texto, com a letra ‘G’, de Gestante, seguida por um algarismo correspondente à ordem das falas: G1...G27.

### Tipo de estudo

Pesquisa qualitativa, de caráter exploratório e descritivo. A sua abordagem possui significados que possibilitam ampliar perspectivas, crenças, convicções e vivências culturais específicas não reduzíveis a variáveis pontuais, lineares e fragmentadas. Seguiu-se os Critérios de Consolidação para Relatórios de Pesquisa Qualitativa (COREQ)^(16)^.

### Procedimentos metodológicos

#### Cenário do estudo

O *corpus* deste estudo foi composto por 27 gestantes, residentes em Aldeias do município de Querência, Mato Grosso, Brasil, o qual possui um total de 31 Aldeias Indígenas, com aproximadamente 5.738 aldeados e especificidades socioeconómicas, raciais, étnicas, culturais e geográficas distintas. As participantes foram acessadas por conveniência, por intermédio das equipes de saúde locais e, após aceite à participação, as mesmas foram contatadas, pessoalmente, pelas pesquisadoras para maiores esclarecimentos e o agendamento de dia e horário para a coleta de dados.

Incluiu-se, no estudo, gestantes indígenas de qualquer uma das 31 aldeias, idade, língua, período gestacional e em condições físicas e emocionais para participarem das entrevistas nos dias e horários previamente agendados. Exclui-se do estudo gestantes de outras línguas desacompanhadas de um interprete (Familiar, Agente Indígena de Saúde, Parteira ou outro). As entrevistas foram audiogravadas com o apoio de um aparelho celular e, na sequência, transcritas para a análise.

#### Coleta e organização dos dados

Os dados foram coletados entre os meses de maio e agosto de 2023, por meio de entrevistas individuais, com duração média de 20 minutos. As entrevistas individuais abertas foram conduzidas com base nos seguintes questionamentos: Fale-me sobre a sua gestação, pré-natal, parto, nascimento e amamentação. Na sua opinião, o que pode ser diferente na assistência às gestantes? Estas questões foram amplamente exploradas, em cada um dos tópicos, de modo a ampliar percepções, vivências e práticas específicas da cultura indígena.

As entrevistas, previamente agendadas, foram conduzidas por um pesquisador com expertise na gestão da saúde indígena e um Enfermeiro colaborador que trabalha nas Aldeias indígenas. Realizou-se, previamente, encontros de gestantes na Secretaria Municipal de Saúde, em sala reservada e ornamentada com objetos artesanais específicos da cultura indígena, tais como: coas, colares, rede, cestas, palhas, moringas e outros e, na sequência, foram realizadas as entrevistas individuais. As entrevistas foram realizadas neste mesmo local, após o encontro de gestantes.

#### Análise dos dados

Os dados foram analisados com base na técnica de análise temática *Reflexive* ^(17)^. Primou-se, nesse processo de análise, pelo significado cultural de cada depoimento e vivência. Buscou-se, para além da contagem do número de informações, a compreensão do sentido existencial e cultural de cada depoimento expresso e/ou não pela gestante indígena.

A análise temática do tipo *Reflexive* foi adotada, neste estudo, a fim de possibilitar o registro sistemático de vivências e *insights,* além de facultar uma codificação espontânea e flexível dos significados singulares atribuídos pelas gestantes indígenas. Assim, essa análise foi sistematizada em seis fases sequenciais e complementares: Familiarização a partir de leituras repetidas dos dados e uma lista rascunhada de ideias; Geração de códigos iniciais, manualmente, pela sistematização de extratos relevantes; Busca de temas a partir da classificação dos diferentes códigos; Refinamento dos temas a partir da validação das temáticas iniciais; Nomeação dos temas a partir da essência que cada tema representa no conjunto de códigos; e a Produção do relatório que ofereceu uma descrição reflexiva do vivido^(16)^, na perspectiva da ODS.

## RESULTADOS

Apresenta-se, inicialmente, a caracterização das participantes do estudo, em relação às variáveis idade, número de consultas de pré-natal, número de gestações, tipo e local do parto, conforme segue na Figura 1.

**Figura 1:** Idade e dados relativos à gestação, pré-natal e parto de mulheres indígenas

A análise dos dados do tipo *Reflexive* resultou em três temáticas reflexivas, quais sejam: Cultuação do parto e nascimento em seu percurso natural e sagrado; Práticas e crenças singulares associadas ao aleitamento materno; Pensamento evoluído ou redutor?

### Cultuação do parto e nascimento em seu percurso natural e sagrado

O parto natural é fortemente desejado e defendido na cultura indígena. Essa prática é reproduzida de mãe para filha e, assim, sucessivamente. Elas reconhecem o corpo como sagrado e, por isso, deve ser livre de cortes ou agressões físicas. Da mesma forma, as “rezas” ou qualquer prática religiosa é aceita e reproduzida como benção e proteção divina. Os “cortes, pernas e braços amarrados” estão associados ao parto hospitalar (cesariana) e possuem a conotação de distanciamento, frieza e violência. Logo, elas desejam parir da forma mais natural possível e junto aos seus familiares.

*Eu quero igual a aldeia, não quero que cortepra meu filho nascer, também não quero que amarrem minhas pernas e braços igual já ouvi falar que faz no hospital.* (G3)

*Eu quero ficar aqui, perto da minha mãe. Não quero que me cortem.* (G5)

*Na aldeia é melhor, porque aqui tem parteira que me ajuda, tem minha mãe que me ajuda também, tem médico e equipe.* (G13)

*A parteira estava lá, ela fez reza e me ajudou para ele nascer, depois ela me ajudou na limpeza e eu fui pra deitar na rede, porque estava sentindo fraqueza.* (G12)

O parto e o nascimento na cultura indígena estão carregados de um misto de sentimentos, rituais e crenças que contribuem e determinam o nascimento em sua forma mais natural possível. Na cultura indígena é respeitado o “tempo do bebê”, independente do tempo de espera para o seu nascimento. O tempo do bebê é respeitado e apreendido como singular e sagrado, ou seja, como percurso inviolável e manipulável por forças externas.

*Édiferente daqui da aldeia. Lá* [hospital] *eles não esperam o tempo do bebê nascer, eles colocam soro pra bebê nascer mais rápido e isso não é normal pra gente, aqui a gente espera o tempo que o bebê vai nascer, isso eu não gostei.* (G5)

*Tive o meu 1º filho com 16 anos, por cesariana. Ele pesou 4kg. O médico dizia que minha barriga era muito grande e que não iria nascer normal. Aí quando comecei com a dor, o médico me disse que iria tirar para sair mais rápido. Foi difícil aceitar.* (G15)

Evidenciou-se, em diversos depoimentos, que mesmo aconselhadas pela equipe de saúde, as gestantes indígenas preferem parir na aldeia, pelo fato de poderem contar com a presença da mãe, da sogra, da parteira da família e outras pessoas próximas.

*Eles nasceram na aldeia, na rede, eu estava com minha mãe e minha sogra que é a parteira. Eu senti muita dor, mas elas me ajudaram.* (G7)

*Na aldeia tem minha mãe, tem minha família, tem parteira e elas ajudam a gente, o bebê foi feito e não vai ficar pra sempre na barriga, uma hora ele vai sair, no tempo certo, e elas ajuda a gente ganhar os bebês na aldeia.* (G11)

*Não quero ir pra cidade* [hospital] *para ter parto. Eu quero ficar aqui com meus filhos e minha família, minha, mãe e minha irmã.* (G24)

Em outros depoimentos, as gestantes indígenas demonstraram que o nascimento no hospital é mais seguro, por vezes, pela indução da equipe de saúde e outras vezes por motivos alheios relacionados a intercorrências obstétricas. Em uma fala, particularmente, evidenciou-se que a gestante queria que o nascimento ocorresse no hospital, embora tenha sido aconselhada a parir na aldeia pelos familiares.

*Eu queria estar morando na cidade pra neném nascer na cidade* [hospital]. *É mais seguro, tem doutor. Eu tenho um filho que é deficiente.* (G1)

*Quero que nasça no hospital, por que lá tem médico, e é melhor, mas minha mãe quer que nasce na aldeia. Aqui na aldeia é muito longe e eu as vezes sinto muita fraqueza.* (G11)

*Quando estava bem, de alimento, de andar a criança pode nascer na aldeia, mas hoje como estou com muita dor, tem dia que a dor vem, e tem dia que ficava muito fraca, aí acha melhor nascer na cidade.* (G16)

*A equipe estava acompanhando na aldeia e eu tive problema de alteração na pressão, o pé e o corpo inchado e muito peso, aí não pode ter parto aqui, aí precisa ter parto no hospital, orientada pelo médico de área e junto com as outras mulheres. A parteira disse que eu tinha que ganhar na cidade e lá o parto não estava evoluindo e precisou fazer cesariana, deu tudo certo.* (G20)

*As duas meninas nasceram na aldeia e os dois meninos nasceram no hospital, porque eu senti dor de infecção de urina e daí não dá para ganhar na aldeia.* (G25)

Em um depoimento, em especial, denotou-se certo empoderamento da gestante em relação à escolha autônoma do tipo de parto, embora tenha ocorrido no hospital. Mesmo sendo no hospital, ela foi acolhida em sua decisão.

*Como já tive cesariana e quis que o segundo filho fosse parto normal, pra não cortar minha barriga, aí precisou ir para a cidade. Quando eu cheguei no hospital eu falei que não queria cesariana, que queria normal. Aí fui pra sala de parto e ele nasceu normal e bem.* (G24)

Apreendeu-se a partir das falas das participantes que o parto natural possui um significado inquestionável na cultura indígena. Nesse percurso é respeitada, acima de tudo, a autonomia da criança, isto é, o seu tempo e a sua hora de nascer, mesmo que a mãe tenha que esperar por muitas horas para o nascimento. A mãe, por sua vez, submete-se às longas horas de dor, por acolher o nascimento como sagrado e inviolável. As práticas religiosas, tais como as rezas, chás, as imposições e outras, adotadas pelas parteiras, se constituem em potente auxílio no alívio das dores do parto.

### Práticas e crenças singulares associadas ao aleitamento materno

As participantes do estudo, na maioria das vezes, reproduziram concepções culturalmente expressas em ambientes indígenas, desprovidas de um pensamento autônomo. Embora muito reduzidas e, por vezes, evasivas, as falas das participantes se limitaram em reproduzir expressões advindas de suas mães ou de alguém ligado à família, como por exemplo: Ela falou que é para o filho ser saudável. (G9) Ela falou que assim fica mais forte. (G15) Ela disse que não pode ser assim. (G17)

A prática do aleitamento materno é inquestionável na cultura indígena. Em momento algum as participantes fizeram referência ao leite fraco ou na impossibilidade de amamentarem. Apenas uma gestante fez referência ao pouco leite, mas que contornou com ervas medicinais específicas. Outra, relatou dor abdominal e necessitou de apoio para conseguir amamentar.

*Ele ainda mama. Tem 2 anos. E agora quando esse outro bebê nascer, um precisa parar.* (G7)

*Todos mamaram. Eu acho que é o que mais importa.* (G9)

*Sim, em um deles eu quase não tinha leite, aí tomei erva e meu leite logo veio. Ele ficou forte, ossos forte, não pega tantas doenças.* (G18)

*Sim, sempre dei peito, uma vez demorou porque minha barriga doía* [cesariana] *e aí minha mãe me ajudava. Fiquei muitos dias com dor, só depois em casa que foi melhor.* (G25)

Evidenciou-se, em grande parte dos depoimentos, que a gestante indígena cessa de amamentar o seu filho, por ocasião de um novo nascimento. Embora a amamentação seja uma prática culturalmente aceita e estimulada, a maioria das mulheres relatou fraqueza, cansaço, desânimo e, em dois casos, a ocorrência de depressão e sem vontade de levantar da cama.

*Eu senti dor, fraqueza. Tem dias que eu não quero levantar, eu e meu esposo a gente já quis ter bebê.* (G2)

*Ela tá cansada de ter bebês. Se queixa muito de fraqueza.* (G5)

*Não quero mais, eu sinto muita fraqueza quando tô gravida. É ruim pra dormir e o bebê mexe muito.* (G11)

Identificou-se, em outros depoimentos, que embora subjugadas em sua cultura, as mulheres indígenas reconhecem as suas forças culturais e as consideram superiores aos dos brancos, especialmente ao compararem o leite materno. Em seu entendimento, o leite artificial está associado à doença conforme segue:

*Porque o leite materno faz a criança ficar bem fortinha, porque o leite dos brancos, de vocês é diferente, porque aí eu não preciso dar o leite dos brancos.* (G11)

*Porque com o leite materno o bebê cresce forte e saudável e na aldeia não tem leite de branco. Eu dei o leite até o outro nascer.* (G16)

*Os outros filhos mamaram no peito. Só um deficiente que usa leite dos brancos.* (G22)

*Sim, porque o leite materno deixa eles bem e eles crescem. O leite que compra, leite de branco é só pra quem tem alguma doença.* (G25)

Na quase totalidade das falas, as participantes são desprovidas de autonomia, ou seja, a autonomia da gestante indígena está atrelada aos processos culturais, integralmente legitimados e aceitos. Logo, o direito de escolha é assegurado pela força da cultura e, sobretudo, pela perpetuação de crenças e práticas religiosas carregadas de sentido em âmbito pessoal e coletivo. Denota-se, assim, um paradoxo entre o que se entende e defende por autonomia na perspectiva intercultural e do pensamento da complexidade.

### Pensamento evoluído ou redutor?

Ao serem questionadas sobre o que poderia ser melhor ou diferente em relação à assistência às gestantes e puérperas, as respostas foram, na sua grande maioria, superficiais e evasivas, mas carregadas de um sentido histórico e cultural.

*Não sei dizer.* (G2, G5, G9, G10, G15, G18, G20)

*Não sei responder.* (G1, G3, G6, G19, G22, G23)

*Não sei.* (G7, G9, G12, G16, G24, G25, G27)

Evidenciou-se, em algumas falas, o desejo de qualificar a assistência pré-natal no âmbito das aldeias, para que não haja a necessidade de transferência para os hospitais, em função das longas distâncias. De modo geral, todas as gestantes almejam parir na aldeia, próximo de suas famílias e, nessa direção, sugerem mais médicos, medicamentos e exames, conforme segue:

*Ter mais exames, ter remédio e médico na aldeia.* (G8)

*Ter vacina, ter remédio pra ajudar. Ter também médico em todas aldeias.* (G11)

*Fazer exames na aldeia e ter mais remédio.* (G13)

*Mais médico e mais equipe em área, para atender nossa família.* (G14)

*Ter mais remédio, mais vitamina para as gestantes e pra quando os filhos ficam doentes.* (G17)

*Ter equipe que fica na aldeia pra quando a gente ter bebê pequeno ajudar quando estão doentes.* (G21)

*Poderia ter parteira para ganhar o bebê na aldeia e o médico também.* (G26)

Embora superficiais e evasivas em suas falas, as participantes do estudo demonstraram vivências e um saber que lhes é singular e, nem por isso, inferior ou superior ao conhecimento de qualquer outra cultura. E, nessa direção, os pesquisadores questionam-se: trata-se de um pensamento evoluído ou redutor? Como dialogar com esta diversidade plural e intercultural de saberes? Onde é preciso evoluir e como evoluir para não incorrer em novas mutilações e alcançar as metas definidas nos ODS?

## DISCUSSÃO

O acesso ao pré-natal e aos cuidados qualificados no parto e puerpério encontram-se intimamente relacionados à organização e a estrutura da Atenção Primária em Saúde, assim como associam-se à redução das desigualdades e à promoção da justiça social. Essas diretrizes aproximam-se, também, dos princípios do Sistema Único de Saúde (SUS), ao conceber a equidade, a universalidade e a integralidade na atenção à saúde como meta para o alcance de melhores resultados em âmbito individual e coletivo^(18)^.

O Ministério da Saúde define que o pré-natal deve ser iniciado até a 12ª semana de gestação, ser composto por, no mínimo, seis consultas de acompanhamento. O pré-natal inadequado ou ineficiente é um fator determinante para desfechos desfavoráveis no parto e nascimento, além de fator de risco para a mortalidade materno-infantil^(19)^, especialmente entre as mulheres indígenas. Assim, a alta fecundidade das mulheres indígenas, representada por intervalos gestacionais curtos e a ocorrência da gravidez na adolescência são, geralmente, fatores que influenciam no ciclo gravídico-puerperal e comprometem a meta prevista na Agenda 2030.

O ciclo gravídico-puerperal abarca, por si só, um misto de sentimentos, crenças e (des)construções, na maioria das vezes não lineares e previsíveis aos olhos da ciência moderna, que necessita respostas, a qualquer custo, para os diferentes eventos existenciais. Na cultura indígena os eventos, mesmo que adversos, ocorrem de forma natural e com especificidades culturais que necessitam ser consideradas, principalmente no parto e nascimento, considerados eventos naturais e sagrados. Qualquer avanço, nessa área, demanda aproximação, diálogo e compartilhamento de vivências culturalmente aceitas e estimadas entre as mulheres indígenas^(20)^.

Evidenciou-se, na fala das participantes deste estudo, um importante paradoxo ao discorrerem sobre a “aldeia indígena”, ambiente que lhes é familiar e acolhedor e o “hospital”, ambiente impessoal, frio e distante, decifrado como cidade. Esse ambiente “hospital” lhes fere o corpo, o sagrado, em decorrência dos cortes da cesariana não desejada pelas gestantes. Esse mesmo ambiente “hospital” lhes distancia da mãe, da sogra, da parteira e de outros familiares que, nas aldeias, participam e celebram o nascimento, considerado um acontecimento coletivo.

Estudos^(6, 21, 22)^ endossam esse pensar das participantes, ao enfatizarem que em muitas culturas indígenas, o nascimento consiste em uma cerimônia religiosa, na qual se celebra uma nova vida, ou seja, um acontecimento que celebra a passagem do mundo espiritual para o mundo físico. As cerimônias de nascimento estão diretamente ligadas à terra, o sol, o céu e, por isso, quanto mais próximo da família e da cultura, tanto mais sagrada e promissora será a saúde e o futuro da criança. Reconhecer o local de nascimento e honrar a sua sacralidade constitui-se em estratégia significativa para assegurar a identidade cultural e implementar os direitos indígenas.

Outro fenômeno singular e complexo está associado ao momento do nascimento, a “hora do bebê” nascer e a “hora da mãe”. Em se tratar de um percurso natural e sagrado, o tempo do bebê sempre é respeitado na cultura indígena, mesmo que a mãe tenha que se submeter a longas horas de espera para o nascimento de seu filho. Nessa relação, a cessaria, por envolver intervenção é vista como violação do sagrado, do “corpo sagrado” que carrega um ser sagrado. O corpo precisa estar livre de intervenções ou interferências externas.

Estudos demonstram, nessa direção, que na cultura indígena as mulheres preferem partos naturais e primam pelas práticas tradicionais e convencionais, isto é, partos nas aldeias e na companhia das famílias. Essas práticas tradicionais favorecem a interação mãe-bebê e reduzem as complicações associadas às rupturas ou infecções perineais. Essas práticas são apoiadas por parteiras que possuem competências baseadas na experiência e no conhecimento adquirido informalmente por meio das tradições e práticas associadas às comunidades indígenas^(23, 24)^.

A teoria indígena possui uma visão de mundo própria, relacionada as tradições e intimamente interconectada com todas as coisas criadas. Fala-se, portanto, de uma teoria holística e abrangente, que envolve passado, presente e futuro e comporta elementos espirituais, emocionais, mentais e físicos do ser e mover-se. Sob esse enfoque, o “hora do nascimento do bebê” é a hora predestinada pelos espíritos do céu e pela Criação^(25, 26)^.

Outro importante paradoxo está relacionado ao “aleitamento materno” e ao “leite dos brancos”. O leite materno é carregado de energia que vincula, fortalece, aproxima e perpetua o sagrado entre as gerações indígenas. Já o “leite dos brancos” está relacionado à doença – criança doente que, por algum motivo, nasceu por cesariana e em ambiente hospital. Estudos demonstram, sob esse enfoque, que cultura indígena o processo saúde-doença está intimamente relacionado ao aleitamento materno imediato e, igualmente, associado ao parto natural nas aldeias, enquanto fenômeno sagrado e protegido^(27, 28)^.

No contexto indígena, a amamentação não figura, apenas, como fonte de alimentação para a criança, mas se constitui em segurança, proteção e conexão entre mãe/família – criança, motivada por valores culturais e espirituais, que aproximam e colocam a criança no centro do processo existencial. Estudos^(29, 30, 31)^ sugerem ampla apreensão destes aspectos culturais, sociais, comunitários e coletivos, no sentido de potencializar as experiências favoráveis de amamentação entre as mulheres indígenas. Para que a amamentação siga sendo uma prática culturalmente aceita é fundamental que se protagonize políticas específicas para as mulheres indígenas, considerando que as mesmas amamentam os seus filhos até o nascimento do próximo filho e, com isso, referem fraqueza, desconforto e mal-estar.

Os resultados deste estudo denotaram que as mulheres indígenas demonstram a necessidade de ampliar a presença de médicos nas aldeias, além do fornecimento de medicamentos e exames, a fim de facilitar o acesso imediato para o atendimento de casos especiais. Evidenciou-se, contudo, em estudos desenvolvidos em outros países, que as mulheres indígenas preferem ser assistidas e orientadas por profissionais de saúde indígena, pela compreensão sólida de práticas culturalmente aceitas e estimuladas^(32, 33)^. Nessa direção, os mesmos estudos demonstram que os agentes de saúde indígena fornecem soluções holísticas e apoio direcionado às necessidades de cada mulher e família.

O ciclo gravídico-puerperal requer, por parte dos profissionais de saúde que atuam em aldeias indígenas, um olhar singular e multidimensional, isto é, tecido por um conjunto de fios/elementos que evocam, no mínimo, mais de uma circunstância ou possibilidade associativa para formar o conhecimento culturalmente significativo^(34)^ e, assim, contribuir para o alcance das metas estabelecidas pela Agenda 2030. É preciso, para tanto, despir-se de percepções preconceituosas e descortinar referenciais que ampliam as perspectivas teóricas e a indução de abordagens que considerem a singularidade e a multidimensionalidade de cada mulher/família indígena.

O ciclo gravídico-puerperal na cultura indígena remonta uma tradição histórico-hegemônica assinalada pelas relações normativas e prescritivas, nas quais o saber científico se sobrepunha à experiência cultural do vivido. Nessa relação, a gestante ou a puérpera indígena era subjugada a um saber biomédico prescritivo, privada de seu saber e significado de vida e cuidado. Esse superpoder hegemônico, consolidado na modernidade, resultou num vazio cultural e dividiu o indivisível e as partes separadas tornando-as sem sentido de vida e sem identidade^(34)^.

Avançar no acesso e na qualidade da assistência às mulheres (gestantes e puérperas) indígenas, requer rupturas de pensamento hegemônicas e a apreensão ampliada e sistêmica das diferentes realidades culturais. (Re)significar e reconectar elementos culturalmente relegados a um segundo plano, requerem um olhar prospectivo por parte dos profissionais da saúde, em geral. A Agenda 2030 e a Agenda de Prioridades do Ministério da Saúde serão alcançados pela retomada de valores e princípios de vida, saúde e bem-estar das diferentes culturas.

### Limitações do estudo

Considera-se como limitação deste estudo a não generalização dos resultados, tendo em vista que as entrevistas foram realizadas com gestantes e puérperas indígenas de apenas uma região do Brasil, o qual apresenta dimensões geográficas continentais e especificidades culturais que variam de aldeia para aldeia indígena.

### Contribuições para a área de Enfermagem e Saúde

As contribuições deste estudo para o avanço da ciência de Enfermagem e Saúde estão associadas à percepção de que é preciso reconectar elementos da cultura indígena, fragmentados e simplificados pela supremacia do saber hegemônico em saúde. Nesse processo de ressignificação, valoração e potencialização de recursos próprios da cultura indígena, o Enfermeiro pode figurar como protagonista e indutor de políticas prospectivas, capazes de contribuir para o alcance dos Objetivos do Desenvolvimento Sustentável.

## CONSIDERAÇÕES FINAIS

As vivências de mulheres indígenas acerca do ciclo gravídico-puerperal são singulares e motivadas por crenças culturais, ambientais e religiosas invioláveis, as quais transcendem o saber científico, as certezas e a linearidade das abordagens contemporâneas, normalmente, instituídas como ordem no contexto da saúde.

O ciclo gravídico-puerperal na cultura indígena precisa ser acolhido e compreendido em suas múltiplas conexões e em associação com os fenômenos ambientais e transcendentais. O parto e o nascimento devem ser considerados como evento natural e em sua dimensão sagrada e coletiva. A prática do aleitamento materno, não se constitui, apenas, em fonte de alimentação para a criança, mas em fonte de energia, segurança, proteção e conexão entre mãe/família – criança.
